# CRISPR/Cas9-mediated knockout of clinically relevant alloantigenes in human primary T cells

**DOI:** 10.1186/s12896-020-00665-4

**Published:** 2021-01-29

**Authors:** Elahe Kamali, Fatemeh Rahbarizadeh, Zohreh Hojati, Morten Frödin

**Affiliations:** 1 Department of Cell and Molecular Biology & Microbiology, Faculty of Biological Science and Technology, University of Isfahan, Isfahan, Iran; 2 Department of Medical Biotechnology, Faculty of Medical Sciences, Tarbiat Modares University, Tehran, Iran; 3 Biotech Research and Innovation Centre (BRIC), Faculty of Health Sciences, University of Copenhagen, Copenhagen, Denmark

**Keywords:** CRISPR/Cas9, Knockout, Genome editing, TCR, T cell

## Abstract

**Background:**

The ability of CRISPR/Cas9 to mutate any desired genomic locus is being increasingly explored in the emerging area of cancer immunotherapy. In this respect, current efforts are mostly focused on the use of autologous (i.e. patient-derived) T cells. The autologous approach, however, has drawbacks in terms of manufacturing time, cost, feasibility and scalability that can affect therapeutic outcome or wider clinical application. The use of allogeneic T cells from healthy donors may overcome these limitations. For this strategy to work, the endogenous T cell receptor (TCR) needs to be knocked out in order to reduce off-tumor, graft-versus-host-disease (GvHD). Furthermore, CD52 may be knocked out in the donor T cells, since this leaves them resistant to the commonly used anti-CD52 monoclonal antibody lymphodepletion regimen aiming to suppress rejection of the infused T cells by the recipient. Despite the great prospect, genetic manipulation of human T cells remains challenging, in particular how to deliver the engineering reagents: virus-mediated delivery entails the inherent risk of altering cancer gene expression by the genomically integrated CRISPR/Cas9. This is avoided by delivery of CRISPR/Cas9 as ribonucleoproteins, which, however, are fragile and technically demanding to produce. Electroporation of CRISPR/Cas9 expression plasmids would bypass the above issues, as this approach is simple, the reagents are robust and easily produced and delivery is transient.

**Results:**

Here, we tested knockout of either TCR or CD52 in human primary T cells, using electroporation of CRISPR/Cas9 plasmids. After validating the CRISPR/Cas9 constructs in human 293 T cells by Tracking of Indels by Decomposition (TIDE) and Indel Detection by Amplicon Analysis (IDAA) on-target genomic analysis, we evaluated their efficacy in primary T cells. Four days after electroporation with the constructs, genomic analysis revealed a knockout rate of 12–14% for the two genes, which translated into 7–8% of cells showing complete loss of surface expression of TCR and CD52 proteins, as determined by flow cytometry analysis.

**Conclusion:**

Our results demonstrate that genomic knockout by electroporation of plasmids encoding CRISPR/Cas9 is technically feasible in human primary T cells, albeit at low efficiency.

## Background

The development of genome editing technologies has enabled site-specific gene disruption or modification at unprecedented efficiencies and holds the promise of treating a broad range of diseases at the genetic level or through engineered cells [1, 2]. Four categories of genome editing systems have been developed: meganucleases, zinc-finger nucleases (ZFNs), transcription activator-like effector nucleases (TALENs), and clustered regularly interspaced short palindromic repeats (CRISPR)/CRISPR-associated (Cas) nucleases [3–6]. These have a common mode of action by binding to a user-defined sequence in genomic DNA and inducing a targeted DNA double-strand break (DSB) [7]. If the DSB occurs in a coding sequence, gene knockout can be achieved by the action of either of two cellular DNA repair pathways; the canonical non-homologous end joining (cNHEJ) pathway and the alternative NHEJ (alt-NHEJ, also known as microhomology-mediated end-joining, MMEJ) pathway [8, 9]. Both pathways typically generate small nucleotide insertion or deletion (indel) mutations at the cleavage site and if the indel disrupts the reading frame, a premature stop codon and functional gene knockout will result. The extent to which either of these repair pathways are utilized depends on the nature of the DSB, the sequence flanking the DSB, the cell type, and the cell cycle stage, where the DSB happens [10–13].

Challenging de novo engineering has impeded wide-spread exploitation of meganucleases, ZFNs and TALENs [14]. By contrast, designing a CRISPR/Cas9 genome editing tool is exceedingly simple: it only involves modifying a 20 nucleotide stretch of the so-called guide (g)RNA component in the system such that it can bind a desired genomic DNA sequence via Watson–Crick base-pairing, thereby directing the associated Cas9 to the target site to elicit a DSB [5, 15, 16].

Adoptive T cell therapy is emerging as a new treatment modality for multiple types of cancers and has produced unprecedented promising results in the clinic [17, 18]. These approaches involve ex vivo manipulation of T cells to express either engineered T-cell receptors (TCRs) or chimeric antigen receptors (CARs) capable of recognizing a particular tumour antigen, or they involve purification and expansion of tumour-infiltrating lymphocytes (TILs) [19]. Most T cell trials have so far used autologous (i.e. patient-derived) T cells. However, while the autologous strategy is simple from the viewpoint of immunogenicity and tolerance, there are substantial obstacles in terms of manufacturing time and expenses, as well as the poor quality and quantity of obtainable T cells, particularly for infants or heavily treated patients. For these reasons, allogeneic (i.e. donor-derived) T cell immunotherapies are currently being explored to circumvent the drawbacks of the autologous-based approach [20]. However, allogeneic T cell transfer also entails several challenges. One problem is that the endogenous TCR present on the infused allogeneic T cells may recognize antigens in the recipient beyond those present on the tumour cells, leading also to off-tumor reactivity, termed graft-versus-host disease (GvHD). Such GvHD could generally be prevented by gene knockout of TCR in the donor T cells [21, 22]. TCR is composed of a TCRα chain, encoded by a single *TRAC* gene, complexed with a TCRβ chain, encoded by two *TRBC* genes. Since the TCRαβ dimer is necessary for full function of TCR, its complete disruption can be achieved by knockout of *TRAC* [7]. Accordingly, the alloreactive potential of donor CAR T cells to elicit GvHD will be eliminated by knocking out their endogenous *TRAC* gene [23]. T cells lacking a TCR component lose CD3 expression and all capabilities for activation via either the CD3 complex or through the TCR. Another problem to be solved is the rejection of infused allogeneic T cells via host-versus-graft (HvG) reactions. It has been proposed that HvG reactions may be prevented by lymphodepleting regimens in the recipient [24], such as alkylating agents and purine nucleotide analogues compounds. Alternatively, lymphodepletion may be achieved using the anti-CD52 antibody alemtuzumab, when combined with knockout of the CD52 gene in the infused T cells, enabling them to evade the lymphodepletion regimen [23].

Given the importance of genomic engineering of human primary T cells for both basic research and immunotherapy, efficient and affordable systems to deliver the engineering reagents, now mainly CRISPR/Cas9, to these cells are critical. Virus-mediated systems and plasmid electroporation are generally the most popular strategies for delivery. Compared with viral-mediated delivery, electroporation of CRISPR/Cas9 expression plasmids is theoretically safer, because it does not entail integration of CRISPR/Cas9 into the genome. Moreover, this method is simpler, faster, and more economical relative to the viral-based delivery system [25].

In this study, we employed transfection of CRISPR/Cas9 plasmid reagents for knockout of *TRAC* and *CD52* genes. We demonstrate that transient expression of these reagents can knockout the TCR complex and CD52 in a human test cell line and in human primary T cells.

## Results

### Generation of CRISPR/Cas9 reagents for knockout of TRAC and CD52

**Fig. 1:**
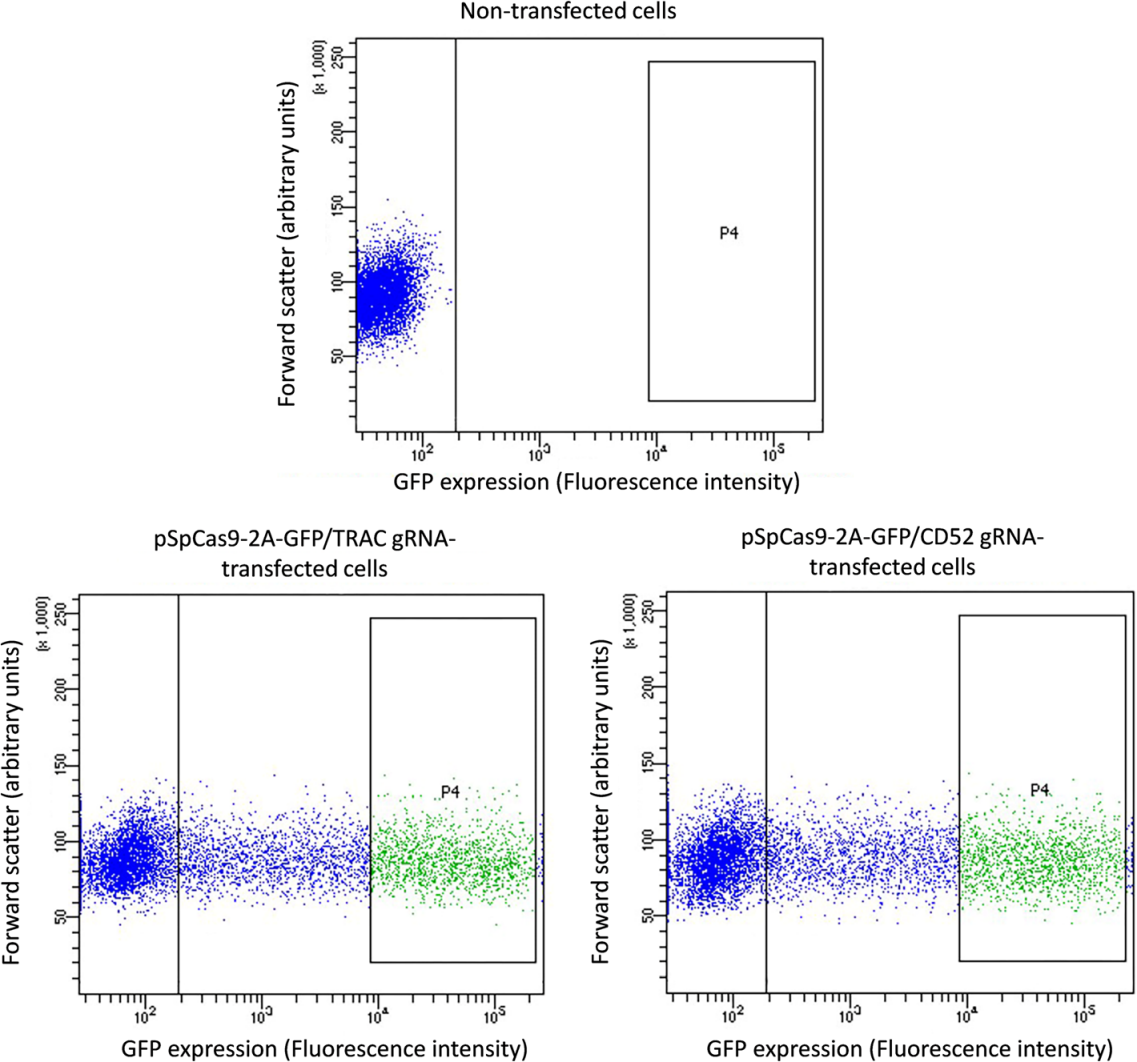
FACS isolation of gRNA/Cas9-expressing 293 T cells. Three days after transfection of 293 T cells with no construct or *TRAC* or *CD52* gRNA-expressing pSpCas9-2A-GFP construct, the cells were subjected to flow cytometry. Analysis of non-transfected cells was used to define the autofluorescence level of the cells in the GFP channel (marked by a bar in the graphs). Next, cells with high GFP expression levels (population 4) were bulk FACS isolated

As the first step towards engineering of human primary T cells, we designed gRNAs targeting exon one of either *TRAC* or *CD52* and introduced them into pSpCas9-2A-GFP plasmid for dual expression of Cas9 and gRNA. We initially tested the ability of the gRNAs to elicit indels in 293 T cells, a cell line frequently used for validation of newly generated CRISPR/Cas9 reagents. Briefly, cells were transfected with gRNA-expressing pSpCas9-2A-GFP plasmids. Three days after transfection, the cells were subjected to FACS to bulk isolate a cell population with high and homogenous gRNA/Cas9 expression, as revealed by the co-expressed GFP marker (population P4 in Fig. 1), which we previously showed translates into high editing levels [26]. Thereafter, genomic DNA was extracted from the FACS isolated cells and indel editing outcomes were evaluated by two different methods that analyse PCR products obtained by amplification of the genomic gRNA target site, i.e. TIDE and IDAA (see Materials and Methods for description of the methods).

**Fig. 2:**
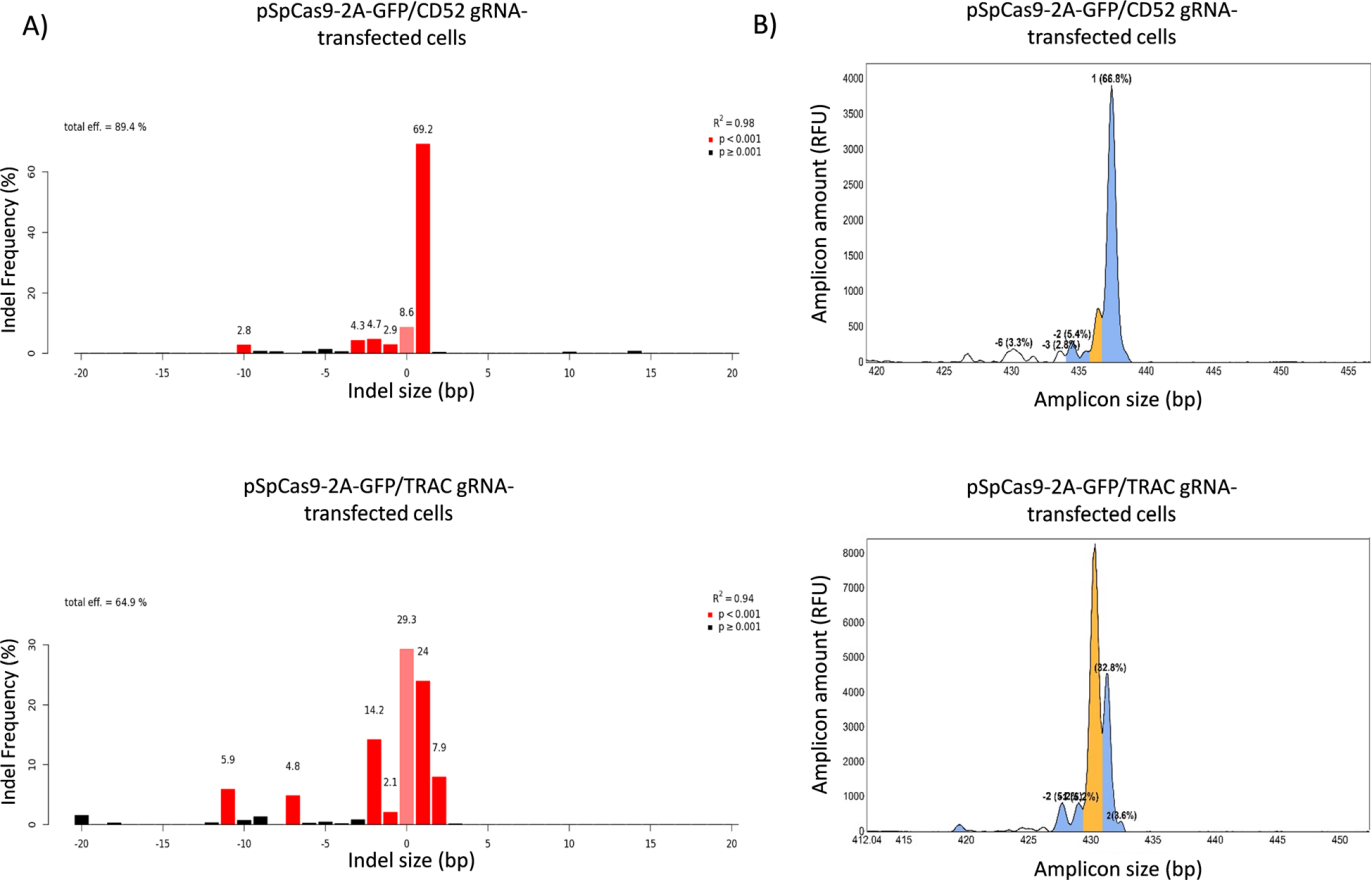
Indel sizes and frequencies elicited by *TRAC* and *CD52* gRNAs in 293 T cells, as analyzed by TIDE and IDAA. The *TRAC* and *CD52* gRNA/Cas9-expressing cell populations FACS-isolated from P4 of Fig. 1 were analyzed by TIDE and IDAA for indels elicited at the respective gRNA target sites. **a**) TIDE shows indels as bars in a graph, indicating indel size in bp on the x-axis and indel frequency on the y-axis. Wild-type allele is indicated by pink color, whereas indels are shown in red (*p*< 0.001) or black (*p*> 0.001), indicating significance of detection. **b**) IDAA shows amplicons derived from the gRNA target sites as peaks, indicating amplicon size in bp, from which indel size can be deduced, on the x-axis and amplicon amount (= indel frequency) on the y-axis. Wild-type allele is indicated by orange color, whereas frame-shifting indels of > 5% frequency are shown in blue and the remainder in white color

For the *CD52* gRNA, TIDE and IDAA revealed essentially the same indel types and extent of editing (Fig. 2 a & b). A 1 bp insertion was the major indel in addition to several low-frequency deletions. Total and frame-shifting (knockout) indels were ~ 89% and ~ 83%, respectively. For the *TRAC* gRNA, the two indel characterization methods also revealed quite similar indel spectra, although a few of the indels were detected with significantly different frequencies. Overall, a 1 bp insertion was a major indel, though less dominant (Fig. 2 a & b). Total and frame-shifting indels were ~ 65% and ~ 62%, respectively. Similar results were obtained in 3 independent experiments. Thus, both gRNAs were able to elicit a high degree of editing as well as knockout editing, as assessed in 293 T cells. Based on comparison with numerous other gRNAs, which we routinely screen in this system and under same conditions, the *CD52* and *TRAC* gRNAs can be categorized as very active.

### Functional inactivation of TCR or CD52 by CRISPR/Cas9 in human primary T cells

**Fig. 3:**
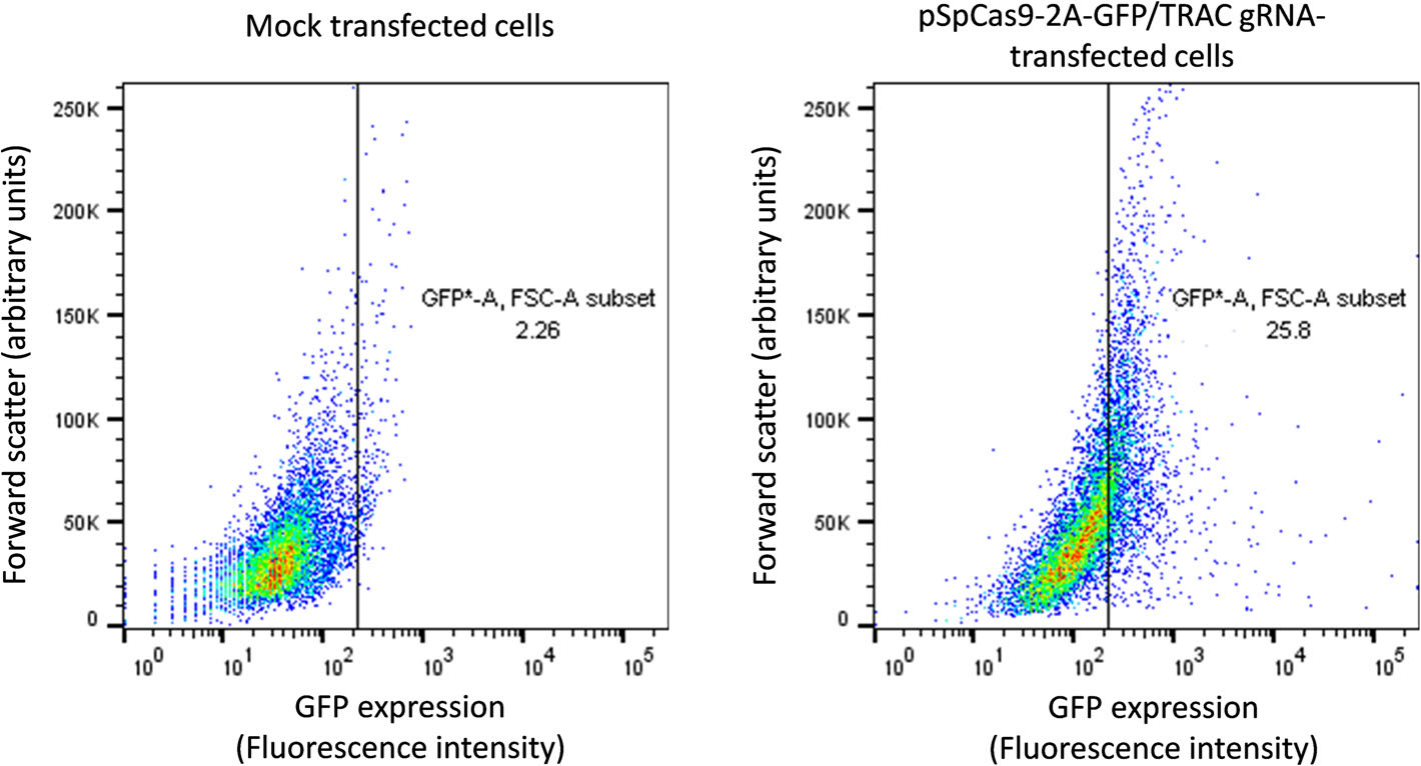
Transfection efficiency of pSpCas9-2A-GFP plasmid in human primary T cells. Three days after electroporation of activated human primary T cells with no plasmid or pSpCas9-2A-GFP/TRAC gRNA plasmid, the cells were analyzed by flow cytometry in the GFP channel. Analysis of the mock transfected cells was used to estimate the autofluorescence level of the cells in the GFP channel (marked by a bar in the graphs). The fraction of the mock transfected cells above this level (~ 2%) was subtracted from the fraction of the pSpCas9-2A-GFP transfected cells above this level (~ 26%), yielding an estimated transfection efficiency of ~ 24%

Having validated that the gRNAs are functional, we proceeded to use them for functional inactivation of TCR and CD52 in activated (proliferating) human primary T cells. For delivery, we chose nucleofection, which is considered the most efficient delivery method of plasmids for these cells. Transfection efficiencies using various plasmid concentrations and electroporation pulse codes were assessed by nucleofection of pSpCas9-2A-GFP/TRAC gRNA plasmid and flow cytometry quantification of GFP positive cells. The transfection efficiencies achieved under the most optimal conditions were around 24% (Fig. 3) and these conditions were chosen for the subsequent experiments.

**Fig. 4:**
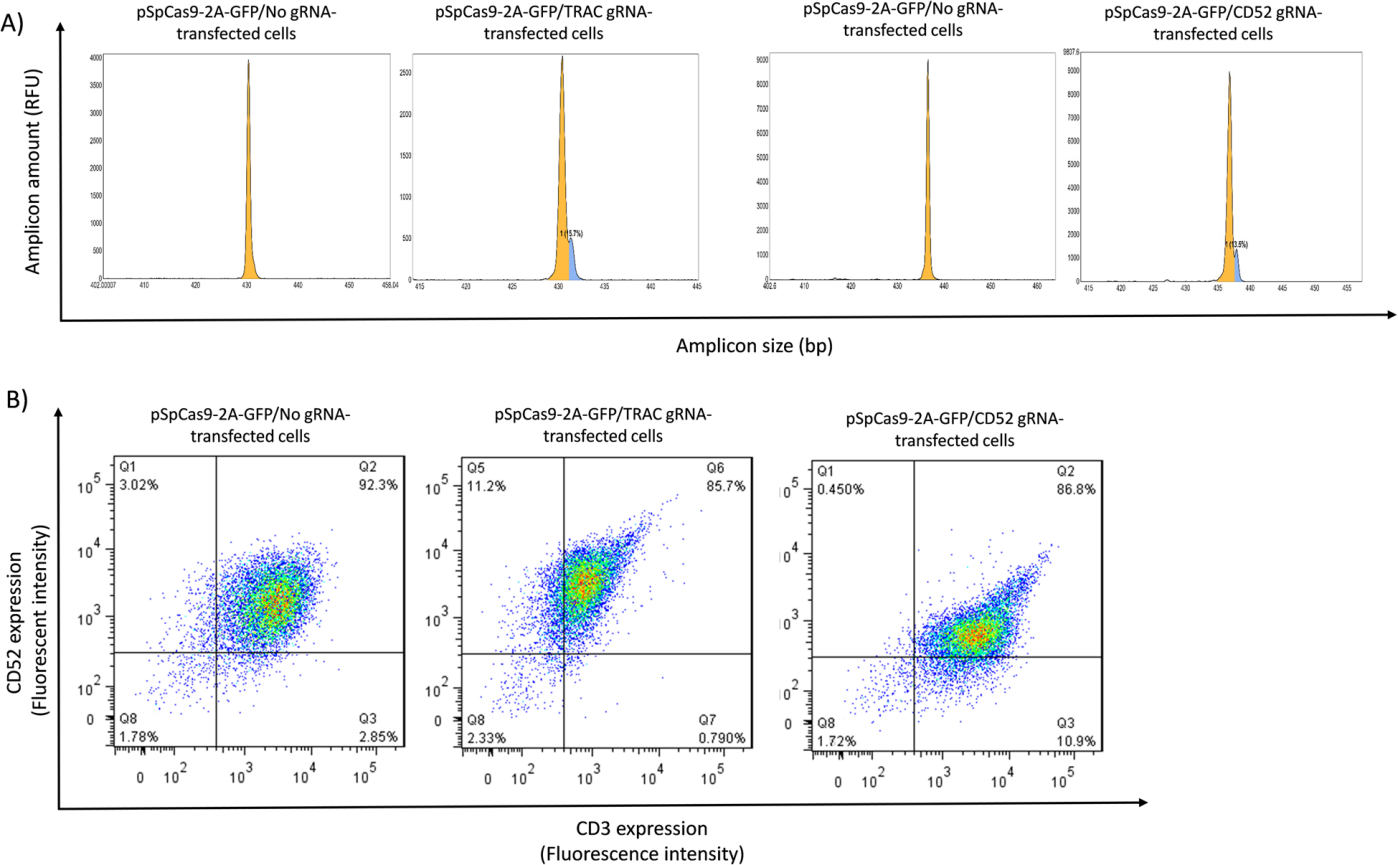
Knockout of *TRAC* and *CD52* in human primary T cells. Four days after transfection of T cells with empty (No gRNA) or *TRAC* or *CD52* gRNA-expressing pSpCas9-2A-GFP construct, the cells were analysed by **a**) IDAA, revealing a 1-bp insertion elicited by both gRNAs and no indels in empty pSpCas9-2A-GFP/No gRNA transfected cells or by **b**) flow cytometry for CD3 (TCR) and CD52 surface expression. This analysis showed an ~ 8% increase of cells in the gates representing background CD3 or CD52 expression levels, indicating that TCR and CD52 knockout was achieved in ~ 8% of the cells

We next nucleofected activated T cells with either empty pSpCas9-2A-GFP/No gRNA plasmid or pSpCas9-2A-GFP plasmid expressing gRNA for either *TRAC* or *CD52*. Four days post-transfection, we purified genomic DNA and analysed the gRNA target sites by IDAA. Strikingly, in T cells, the mixed indel spectrum observed for both the *TRAC* and *CD52* gRNAs in 293 T cells was replaced by one single 1-bp insertion (Fig. 4a). The mean frequencies and S.D. of this frameshifting indel were 14.3% +/− 3% for the *TRAC* gRNA and 12.5% +/− 1.4% for the *CD52* gRNA, as determined in 3 experiments. Note that no indels at the *TRAC* or *CD52* gRNA target sites were observed in cells transfected with empty pSpCas9-2A-GFP/No gRNA plasmid, demonstrating that the editing was specific.

Finally, we quantified the cell surface levels of the targeted proteins on the edited T-cells by flow cytometry. To quantify TCR expression, we used a specific monoclonal antibody to monitor CD3ε, which is only present on the T cell surface, when TCRαβ are expressed [27]. CD52 expression was quantified using the anti-CD52 monoclonal antibody, from which alemtuzumab is derived. As illustrated in Fig. 4b, flow cytometry revealed that the *TRAC* gRNA increased the fraction of cells with background levels of CD3 from ~ 3% (Q1 in left panel) to ~ 11% (Q5 in middle panel), suggesting functional knockout of TCR in ~ 8% of the T cell population (11–3%=8%). Similarly, the *CD52* gRNA increased the fraction of cells with CD52 back ground levels from ~ 3% (Q3 in left panel) to ~ 11% (Q3 in right panel), suggesting functional knockout of CD52 in ~ 8% of the T cell population (11–3%=8%). The mean knockout frequencies and S.D. of similar quantifications were 7.1% +/− 1% for the *TRAC* gRNA and 7.8% +/− 0.3% for the *CD52* gRNA, as determined in 3 experiments.

## Discussion

In this study, we exploited the CRISPR/Cas9 gene editing platform to knockout two clinically relevant genes, *TRAC* and *CD52,* in human primary T cells. Our experimental workflow comprised isolation and activation of human primary T cells followed by electroporation-based transfer of plasmids expressing Cas9 and gRNAs specific for the *TRAC* and *CD52* genes. Prior to testing in primary T cells, the activity of each gRNA was validated in the 293 T cell line.

The ability of both gRNAs to elicit indels (i.e. activity), as tested in 293 T cells could be extrapolated to primary T cells. However, the indel spectrum was different, as the bias towards a 1-bp insertion observed in 293 T cells was a complete predominance of the 1-bp insertion in T cells. One-bp insertions are elicited by the NHEJ pathway, which operates throughout the cell cycle, whereas indels ≥2 bp are typically the result of MMEJ repair, which is restricted to S-phase and G2 of the cell cycle [8, 9, 28, 29]. The observed shift therefore most likely reflects a more pronounced cell cycle arrest in the primary cells, as compared to the cell line. This may be due to the fact that the primary cells are more prone to p53-mediated cell cycle arrest in response to the Cas9-elicited DSB, as compared to the cell line [30] or that they are more sensitive to the toxic effects of the transfected plasmid DNA [31, 32]. Regardless of the mechanism, the data highlights that it is important to determine the indel spectrum of gRNAs for T cell editing in T cells, since indel spectra obtained in other cell types may be different. In the present case, our indel analyses demonstrated that both gRNAs elicited frame-shifting, i.e. functional knockout indels in human primary T cells. Accordingly, both gRNAs were also found to decrease TCR (CD3) and CD52 surface expression to background levels in 7–8% of the transfected T cells, which probably reflects knockout of both alleles in this cell population. A presumed, biallelic knockout of 7–8% is in good agreement with the observed 12–14% total frameshift indel mutagenesis, as biallelic:monoallelic indel modification often occur in 1:2–1:3 ratios in CRISPR/Cas9 editing.

To date, several attempts have been reported, which combine knockout of the endogenous TCR, using ZFNs [2, 21], TALENs [22, 23, 33] or CRISPR/Cas9 [7, 34, 35], with redirecting of T cells for cancer immunotherapy. In early efforts of generating allogeneic T cells, ZFNs and TALENs were used to inactivate the TRAC or TRBC genes [21, 22]. Both studies used mRNA electroporation-based protocols and achieved gene disruption rates of 20–30% for ZFNs [21] and 40–60% for TALENs [2]. In another study, electroporation of TALEN mRNAs were used to target TRAC and CD52 in CD19 CAR-T cells with disruption rates of > 70 and > 60%, respectively ‌ [23]. This study also demonstrated in vivo anti-tumor activity of the TCR/CD52-deficient CAR-T cells in a lymphoma murine model.

Now, CRISPR/Cas9 has become the genome editing technology of choice, and three major delivery systems have been developed: viral, plasmid and RNP. Early attempts to apply viral “all-in-one” CRISPR/Cas9 delivery systems in human primary T cells resulted in low targeting efficiencies [36, 37]. No attempts to increase the efficiencies have subsequently been reported, most likely due to the inherent risks associated with genomic integration of the editing tools. Plasmid based delivery has gained widespread use in the general editing community due to its many advantages and has therefore also been explored for T cell editing. The advantages include transient and therefore, in theory, safer delivery, ease of use, versatility, typically robust efficiencies and high stability as well as ease and affordability regarding production [38]. Moreover, thousands of CRISPR-related products are currently available as plasmids and a large knowledge base on plasmid-mediated CRISPR/Cas9 editing has been developed.

Unfortunately however, there is growing evidence that DNA-based approaches work inefficiently for CRISPR/Cas9 gene editing in primary T cells [34, 39, 40]. In one report, gRNAs exhibiting substantial efficiency in 293 T cells showed low or no significant efficiency in primary T cells [31], in agreement with a subsequent study showing that high editing of plasmid-based CRISPR/Cas9 in cell lines may not translate into high editing activity in primary cells [40]. Our present work supports the notion of relatively inefficient editing of CRISPR/Cas9 plasmids in T cells. There may be several reasons for this, among which, relatively low transfection efficiencies represent one limiting factor, as also illustrated by our study. Thus, despite using nucleofection, which generally is the most efficient transfection approach for hard-to-transfect cells, we only achieved relatively modest delivery to the T cells. DNA toxicity is another likely obstacle for the use of plasmid-based CRISPR/Cas9 editing in T cells [31, 41], in part due to the innate immune response of T cells to double-stranded nucleic acids [42]. Looking forward, it is possible that the low efficiencies of CRISPR/Cas9 plasmids in T cells may be elevated by FACS-based genome editing: such approaches may enable isolation of a population of cells that all express CRISPR/Cas9, but at a defined and relatively low level that minimizes toxicity, yet allows sufficient editing, as has been described for other cell types [26].

The third, and more recently developed, RNP gene editing system is highly-priced, precluding exploitation by low-budget research groups, and the reagents are very fragile. However, RNPs seem to overcome the shortcomings described above for CRISPR/Cas9 plasmids: thus, they are appearing very efficient and do not pose the toxicity associated with plasmids [39, 43, 44]. Furthermore, they have high amenability to multiplex genome editing [43]. Altogether, RNPs are therefore emerging as the so far most promising method for CRISPR/Cas9-based T cell.

## Conclusion

In conclusion, our data demonstrate that genomic knockout using CRISPR/Cas9 plasmids expressing sgRNA and Cas9 is technically feasible in human primary T cells, however, at relatively low efficiencies, which will hamper its clinical application, where high efficiency will be desirable. Future efforts would need to solve the issues causing low efficiencies of CRISPR/Cas9 plasmids in T cells or alternatively, continue the development of the efficient RNP-based platform in primary T cells to provide a high-throughput method for therapeutic applications.

## Materials and methods

### Guide RNA design and plasmid construction

The gene sequences of human *TRAC* and *CD52* genes were downloaded from NCBI and ensemble websites and gRNAs were designed to target the first exon of each gene using the CRISPR Design tool (http://crispr.mit.edu). For each gRNA, two complementary 5′-phosphorylated oligonucleotides encompassing the gRNA sequence and BbsI restriction endonuclease site overhangs were synthesized (Table 1), annealed and sub-cloned into pSpCas9-2A-GFP plasmid (pX458, Addgene 48,138#, Cambridge, MA, USA) that had been digested with BbsI (New England Biolabs, Beverly, MA, USA), using a Golden Gate assembly cloning strategy [45], and gel purified using a Gel Extraction Kit (Qiagen). The resultant constructs were subjected to Sanger sequencing to verify proper sub-cloning of the gRNA sequences.

**Table 1:** Oligos used for introduction of gRNA sequences for *TRAC* and *CD52* genes into pSpCas9-2A-GFP plasmid

Name	Sequence
TRAC-gRNA (F/top) TRAC-gRNA (R/bottom)	5′p-CACCGTCTCTCAGCTGGTACACGGC-3′ 5′p-AAACGCCGTGTACCAGCTGAGAGAC-3′
CD52-gRNA (F/top) CD52-gRNA (R/bottom)	5′p-CACCGCAGCCTCCTGGTTATGGTAC-3′ 5′p-AAACGTACCATAACCAGGAGGCTGC-3′

The gRNA sequences are underlined

### Cell culture

The human embryonic kidney (HEK) 293 T cell line (ATCC, cat. no. CRL-11268) was maintained in Dulbecco’s Modified Eagle’s Medium (high glucose). Peripheral blood mononuclear cells were isolated from blood (buffy coats) of healthy volunteer donor (following written informed consent) using Ficoll-Paque density gradient. CD3^+^ T cells were isolated from peripheral blood mononuclear cells using the Pan T Cell Isolation Kit (Miltenyi Biotec, Bergisch Gladbach, Germany) according to the manufacturer’s guidelines. T cells were expanded through activation by anti-human CD3/CD28 magnetic dynabeads (Thermo Fisher Scientific, Inc., Waltham, MA, USA) at a bead to cell ratio of 1:1 and 30 IU/mL of recombinant human IL-2 (Miltenyi Biotec, Bergisch Gladbach, Germany) in RPMI 1640 medium (Gibco; Thermo Fisher Scientific, Inc., Waltham, MA, USA) supplemented with Glutamax (Gibco; Thermo Fisher Scientific, Inc., Waltham, MA, USA) and 1 mM sodium pyruvate. All media were supplemented with 0.1 mg/mL Penicillin and Streptomycin (Gibco; Thermo Fisher Scientific, Inc., Waltham, MA, USA), and 10% Fetal Bovine Serum (Gibco; Thermo Fisher Scientific, Inc., Waltham, MA, USA). All cells were cultured at 37 °C in humidified atmosphere with 5% CO2.

### 293 T cell transfection

Plasmids were introduced into ~ 80% confluent 293 T cells in 24-well (2 cm^2^) plates using Lipofectamine™ 3000 (Life Technologies, San Diego, CA, USA) according to the manufacturer’s instructions. Briefly, 0.5 μg of gRNA-expressing pSpCas9(BB)-2A-GFP plasmid construct was diluted in 25 μL Opti-MEM reduced serum media (Gibco; Thermo Fisher Scientific, Inc., Waltham, MA, USA) supplemented with 1 μL of P3000 Reagent (Life Technologies). Then, 1.5 μl Lipofectamine 3000 reagent was diluted in 25 μL Opti-MEM and thereafter mixed with the diluted DNA/P3000 Reagent. The mixtures were incubated at room temperature for 5 min and thereafter added dropwise to each well of cells.

### Primary T cell transfection

Three days after activation of the T cells, the CD3/CD28 Dynabeads were magnetically removed and T cells were cultured in the absence of beads for 6–12 h and thereafter electroporated using the P3 Primary Cell 4D-Nucleofector™ X Kit S, (V4XP-3032, Lonza, Cologne, Germany), according to the following protocol: briefly, 1× 10^6^ cells were washed twice with phosphate-buffered saline by centrifuging at 300 *g* for 5 min and thereafter resuspended together with the gRNA-expressing pSpCas9-2A-GFP plasmid in nucleofection buffer. The resulting cell mixture was transferred to nucleofection cuvette and immediately subjected to nucleofection in an Amaxa 4D-Nucleofection device, using program EH-115. After nucleofection, the cell mixture was gently transferred to a dish with pre-warmed medium and incubated at 37 °C.

### Fluorescence activated cell sorting (FACS) of GFP-expressing 293 T cells

Three days after transfection, 293 T cells were subjected to FACS isolation of cells with high levels of GFP, encoded by pSpCas9-2A-GFP, and consequently high levels of editing, essentially, using procedures we described previously [26]. Briefly, the cells were detached using trypsin, then passed through a 50-μm filcon (BD Biosciences, San Jose, CA) to achieve a single-cell suspension and finally sorted for a desired GFP fluorescence level in a BD FACS Aria™ III instrument (BD Biosciences, San Jose, CA), using non-transfected cells to define the background fluorescence level. After FACS isolation, cells were harvested.

### Evaluation of indel mutagenesis

The CRISPR/Cas9-induced indel frequencies were quantified by Indel Detection by Amplicon Analysis (IDAA), using procedures we described previously [26] and by Tracking of Indels by Decomposition (TIDE), as described by others [46]. IDAA is based on PCR amplification of the gRNA target site of the edited sample using a tri-primer amplicon fluorescence labeling set-up that allows determination of amplicon sizes and frequencies by subsequent DNA fragment analysis and quantification by capillary electrophoresis in a sequenator [26]. Amplicons containing a deletion or an insertion will be shorter or longer than amplicons derived from the wild-type allele, thereby allowing determination of indel sizes, and the relative indel frequencies are reflected by peak size. TIDE analysis is based on two PCR amplifications of the gRNA target site, performed on the edited sample and a wild-type (unedited) control sample. The PCR products are next subjected to standard Sanger sequencing and the two sequence data files are uploaded to the TIDE webtool (http://tide.nki.nl). The sequencing traces are then analyzed using a specially developed algorithm, which is provided as an easy-to-use web tool that determines the indels present in the edited sample and quantifies frequencies by computational decomposition of the mixture of sequence traces in the edited sample relative to the control sample.

Briefly, genomic DNA was first extracted from cells using QuickExtract buffer (Epicentre, Madison, WI, USA), yielding lysates of 3000–4000 cells/μl that were incubated at 65 °C for 20 min, followed by 98 °C for 10 min. For IDAA, the tri-primer set-up encompassed: a gene-specific forward primer with a common 5′ overhang, a gene-specific reverse primer, and a universal 6-FAM 5′-labelled forward primer (FamFwd) which has same sequence as the overhang on the gene-specific forward primer (Table 2). PCR amplification was done using TEMPase Hot Start DNA Polymerase (Amplicon, Denmark), 10:1:10 ratio of FamFwd:Fwd:Rev primers and touchdown thermocycling conditions: an initial 72 °C annealing temperature ramping down by 1 degree/cycle to 58 °C, followed by an additional 25 cycles using 58 °C annealing temperature. Dilutions of the PCR products were mixed with GS500LIZ size standard (Thermo Fisher Scientific) and applied to fragment analysis on an ABI3500 sequenator (ABI/Life Technologies, USA) according to the manufacturer’s instructions. Data were analyzed using Viking ProfileIt™ indel profiling software (https://viking.sdu.dk).

**Table 2:** IDAA primers

Primer name	Sequence (5′ to 3′)
IDAA-CD52-Fwd IDAA-CD52-Rev	5′-*AGCTGACCGGCAGCAAAATTG*TGTACGATCTAGCCTGCTCC-3′ 5′-AGTCCATCTGCTGTGCTCTC-3′
IDAA-TRAC-Fwd IDAA-TRAC-Rev	5′-*AGCTGACCGGCAGCAAAATTG*TCACGAGCAGCTGGTTTCTA-3′ 5′-ATGCTGTTGTTGAAGGCGTT-3′

For TIDE, purified PCR products amplifying the gRNA target sites from edited and wild-type samples were Sanger-sequenced and analyzed with the TIDE webtool (http://tide.nki.nl).

### Flow Cytometry

TCR and CD52 surface disruption was quantified using flow cytometry analysis 4 days post-electroporation. Transfected primary T cells were thoroughly washed with phosphate-buffered saline containing 1% Fetal Bovine Serum and stained with mouse APC-conjugated anti-human CD3 (BD Biosciences) or PE-conjugated anti-human CD52 (BD Biosciences) for 20–30 min at 4 °C in dark. As isotype controls were used mouse APC-conjugated IgG1κ (BD Biosciences) or mouse PE-conjugated IgG3κ (BD Biosciences). A BD FACSCalibur™ instrument (BD Bioscience) was used to perform the flow cytometry analysis. All data were analyzed using FlowJo Software.

## Data Availability

The data that support the findings of this study are available from the corresponding authors upon reasonable request.

## Abbreviations

CRISPR: clustered regularly-interspaced short palindromic repeats
TCR: T cell receptor
CAR: chimeric antigen receptor
GvHD: graft-versus-host-disease
ZFN: zinc-finger nucleases
TALEN: transcription activator-like effector nuclease
NHEJ: non-homologous end joining
MMEJ: microhomology-mediated end-joining
TIL: tumour-infiltrating lymphocytes
DSB: double-strand break
InDel: insertion or deletion
HvG: host-versus-graft
IDAA: Indel Detection by Amplicon Analysis
TIDE: Tracking of Indels by Decomposition
HEK: human embryonic kidney
FACS: Fluorescence Activated Cell Sorting
